# A novel web-based risk calculator for predicting surgical site infection in HIV-positive facture patients: a multicenter cohort study in China

**DOI:** 10.3389/fcimb.2024.1408388

**Published:** 2024-06-26

**Authors:** Bo Liu, Guo Wei, Liqiang Hu, Qiang Zhang

**Affiliations:** ^1^ Department of Orthopaedics, Beijing Ditan Hospital, Capital Medical University, National Center for Infectious Diseases, Beijing, China; ^2^ Department of Surgery, Chengdu Public Health Clinical Medical Center, Chengdu, China; ^3^ Department of Orthopaedics, Changsha First Hospital, Xiangya Medical College, Changsha, China

**Keywords:** HIV, fracture, surgical site infection, binary logistic regression, SVM-RFE, predictive model, multicenter cohort study, risk factors

## Abstract

**Background:**

Surgical site infection (SSI) is a common complication in HIV-positive fracture patients undergoing surgery, leading to increased morbidity, mortality, and healthcare costs. Accurate prediction of SSI risk can help guide clinical decision-making and improve patient outcomes. However, there is a lack of user-friendly, Web-based calculator for predicting SSI risk in this patient population.

**Objective:**

This study aimed to develop and validate a novel web-based risk calculator for predicting SSI in HIV-positive fracture patients undergoing surgery in China.

**Method:**

A multicenter retrospective cohort study was conducted using data from HIV-positive fracture patients who underwent surgery in three tertiary hospitals in China between May 2011 and September 2023. We used patients from Beijing Ditan Hospital as the training cohort and patients from Chengdu Public Health and Changsha First Hospital as the external validation cohort. Univariate, multivariate logistic regression analyses and SVM-RFE were performed to identify independent risk factors for SSIs. A web-based calculator was developed using the identified risk factors and validated using an external validation cohort. The performance of the nomogram was evaluated using the area under the receiver operating characteristic (AUC) curves, calibration plots, and decision curve analysis (DCA).

**Results:**

A total of 338 HIV-positive patients were included in the study, with 216 patients in the training cohort and 122 patients in the validation cohort. The overall SSI incidence was 10.7%. The web-based risk calculator (https://sydtliubo.shinyapps.io/DynNom_for_SSI/) incorporated six risk factors: HBV/HCV co-infection, HIV RNA load, CD4+ T-cell count, Neu and Lym level. The nomogram demonstrated good discrimination, with an AUC of 0.890 in the training cohort and 0.853 in the validation cohort. The calibration plot showed good agreement between predicted and observed SSI probabilities. The DCA indicated that the nomogram had clinical utility across a wide range of threshold probabilities.

**Conclusion:**

Our study developed and validated a novel web-based risk calculator for predicting SSI risk in HIV-positive fracture patients undergoing surgery in China. The nomogram demonstrated good discrimination, calibration, and clinical utility, and can serve as a valuable tool for risk stratification and clinical decision-making in this patient population. Future studies should focus on integrating this nomogram into hospital information systems for real-time risk assessment and management.

## Introduction

HIV (Human Immunodeficiency Virus) continues to be a major global health challenge, affecting millions of individuals worldwide. According to the latest statistics from UNAIDS, an estimated 39 million [33.1 million–45.7 million] people were living with HIV globally in 2022 (Global HIV & AIDS statistics—Fact sheet). Despite remarkable progress in the development and widespread use of antiretroviral therapy (ART), which has greatly improved the prognosis and life expectancy of HIV-positive individuals, this population continues to face a myriad of health challenges (de Los Rios et al., 2021). ART has transformed HIV from a once fatal disease into a manageable chronic condition, enabling HIV-positive individuals to live longer and healthier lives. However, the persistent immunodeficiency and chronic inflammation associated with HIV infection render this population more susceptible to various opportunistic infections and increase their risk of surgical complications (Narayan et al., 2014). As the life expectancy of HIV-positive individuals continues to improve, thanks to advancements in ART and comprehensive HIV care, the demand for surgical interventions in this population is also growing (Deeks et al., 2013). Healthcare providers must be equipped with the knowledge and tools to provide safe and effective surgical care to HIV-positive patients while minimizing the risk of complications. This necessitates a comprehensive understanding of the complex interplay between HIV infection, immunodeficiency, ART, and surgical outcomes. By addressing these challenges and optimizing perioperative management strategies, we can improve surgical outcomes and quality of life for HIV-positive individuals.

Surgical site infections (SSIs) is one of the most common and serious complications following surgical procedures, leading to prolonged hospital stays, increased healthcare costs, and higher morbidity and mortality rates (Berríos-Torres et al., 2017). SSIs can cause significant pain and discomfort for patients, delay wound healing, and require additional antibiotic treatment or surgical interventions. In the general population, SSIs occur in 2–5% of patients undergoing surgical procedures. However, HIV-positive patients are particularly vulnerable to SSIs due to their compromised immune system and altered wound healing processes (Muchuweti and Jönsson, 2015). The impaired immune function in HIV-positive individuals can lead to decreased inflammatory response, reduced chemotaxis of immune cells, and impaired phagocytosis, all of which contribute to an increased risk of SSIs. Several studies have investigated the incidence and risk factors of SSIs in HIV-positive patients undergoing various surgical procedures. Zhang et al. (2012) reported The incidence rate of SSI among HIV-infected patients has been reported to be 47.5%, with types of SSIs including 38.4% incisional SSIs, 5.4% deep incisional SSIs, and 3.7% organ/space SSIs. For instance, a systematic review and meta-analysis found that the pooled risk ratio of infection in HIV patients compared to non-HIV patients was 1.8, indicating a higher risk of post-operative SSIs among HIV-infected patients (Kigera et al., 2012). The study by Abalo et al. indeed sheds light on the heightened risk of surgical site infections (SSIs) among HIV-positive patients undergoing orthopedic surgery. Their research identified an incidence rate of 20.8% for SSIs in this specific patient population, which is notably higher compared to the rates typically reported in the general population (Abalo et al., 2010). These findings underscore the urgent need to develop targeted strategies for preventing and managing SSIs in HIV-positive patients. Numerous risk factors have been identified for SSIs in HIV-positive patients, including low CD4 count, high viral load, malnutrition, and co-existing infections (Abalo et al., 2010; Zhang et al., 2012; Korol et al., 2013). Understanding and addressing these risk factors is crucial for developing effective SSI prevention and management strategies in HIV-positive patients.

Some predictive models for SSI have been developed to assist in risk stratification and clinical decision-making, these models typically incorporate various patient-, surgery-, and hospital-related factors to estimate the probability of SSI occurrence (Troillet et al., 2017). However, most existing models are based on general surgical populations and may not adequately capture the unique risk factors and characteristics of HIV-positive patients (Alexander et al., 2011). For example, Morales et al. (2011), focused on assessing the application of the National Nosocomial Infections Surveillance (NNIS) and Efficacy of Nosocomial Infection Control (SENIC) indexes in patients undergoing surgery for abdominal trauma and aimed to develop an alternative model to predict surgical site infections (SSIs), however, it does not include HIV-specific parameters, such as CD4 count or viral load, which are critical in assessing SSI risk in this population. Additionally, Srun et al. (2013), evaluated the applicability of the US National Nosocomial Infections Surveillance (NNIS) risk index in an Australian setting for various outcomes of surgical site infection (SSI), these models often rely on complex algorithms and require manual data input, limiting their usability and accessibility in real-world clinical settings. The study found that the sensitivity of the NNIS risk index was low for all SSI outcomes, ranging from 0.47 to 0.69 and from 0.09 to 0.20 using NNIS risk index thresholds of 1 and 2, respectively. The complex nature of these models can hinder their widespread adoption by healthcare providers, particularly in resource-limited settings where HIV prevalence is high. To address these limitations, there is a pressing need for a novel, web-based predictive model specifically tailored for HIV-positive patients, which can provide accurate and user-friendly SSI risk assessment.

The primary objective of this study is to develop a novel, web-based predictive model specifically designed for assessing the risk of surgical site infection (SSI) in HIV-positive patients. To validate the performance of the newly developed SSI predictive model, we will conduct a multicenter cohort study involving HIV-positive patients undergoing surgical procedures. This model will incorporate key risk factors and clinical parameters relevant to the HIV-positive population, aiming to provide more accurate and personalized risk predictions compared to existing generic models. By leveraging web-based technologies, the model will offer a user-friendly interface and facilitate easy access for healthcare professionals, ultimately promoting its adoption in clinical practice. The development and validation of such a model would represent a significant advance in the field of HIV and surgery, addressing a critical unmet need and potentially transforming the care of this vulnerable population.

## Methods and materials

### Study design and participants selection

**Figure 1:**
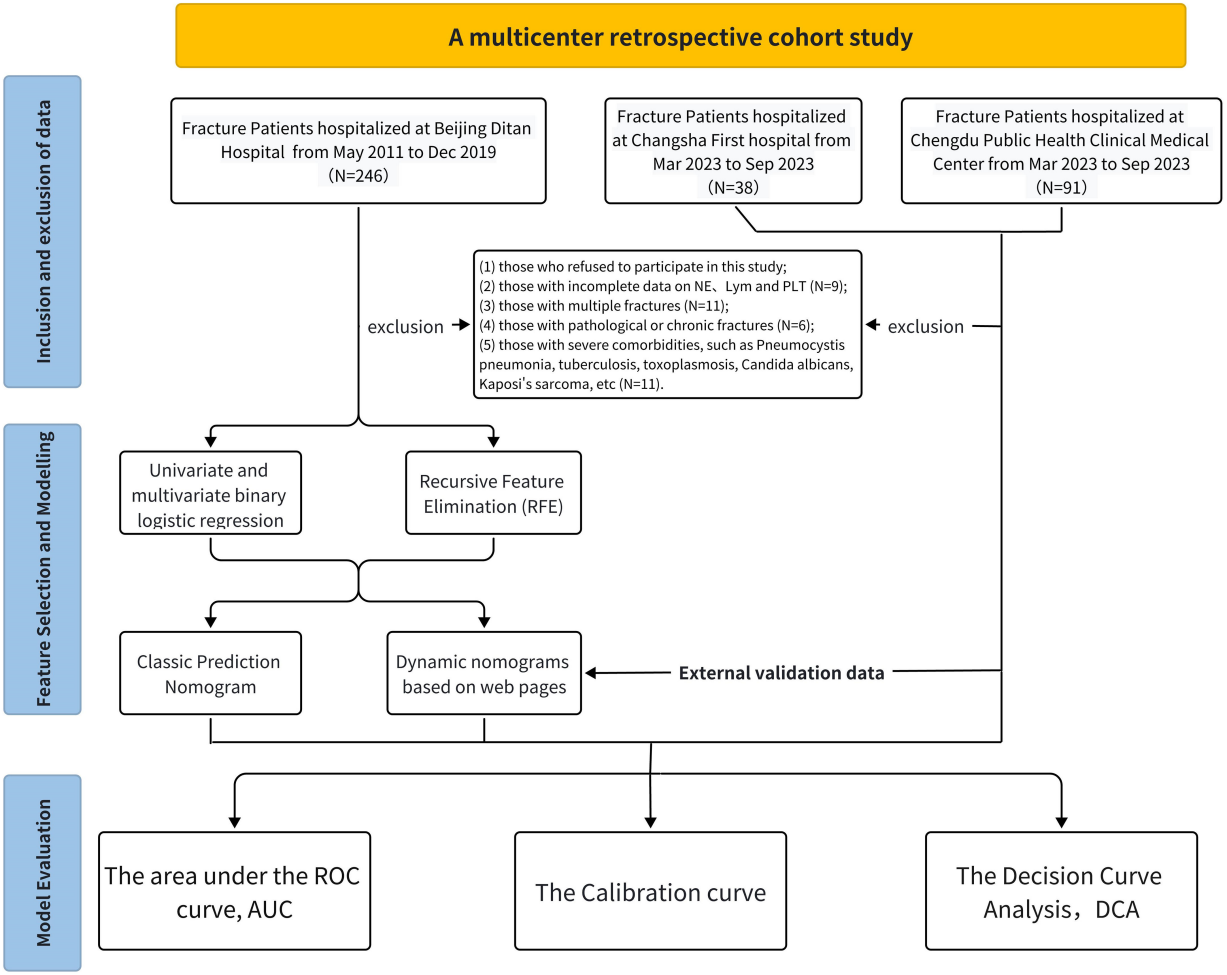
Flow chart of our study.

This multicenter retrospective cohort study aimed to collect and analyze data from HIV-positive patients diagnosed with fractures across three healthcare facilities: Beijing Ditan Hospital, Changsha First Hospital, and Chengdu Public Health Medical Center. Among them, patient data from Beijing Ditan Hospital, National Center for Infectious Diseases, were used for training cohort; data from Chengdu Public Health Clinical Medical Center and Changsha First Hospital were used as external validation datasets (**Figure 1**).

Inclusion criteria encompassed: 1) Patients admitted to the Orthopedics Department of Beijing Ditan Hospital from May 2011 to December 2019; 2) Patients hospitalized in the Orthopedics Department of Changsha First Hospital from March to September 2023; 3) Patients admitted to the Orthopedics Department of Chengdu Public Health Medical Clinical Center during the same period; 4) HIV-positive individuals diagnosed with fractures; 5) Patients undergoing surgical intervention; 6) Those who received follow-up examinations within 3 to 12 months post-surgery; 7) Patients aged between 19 and 85 years.

Exclusion criteria included: 1) Individuals who declined participation (n=3); 2) Patients with incomplete neutrophil, lymphocyte, and platelet data (n=6); 3) Those with multiple fractures (n=11); 4) Patients with pathological or old fractures (n=6); 5) Individuals with severe comorbidities such as Pneumocystis pneumonia, tuberculosis, toxoplasmosis, Candida albicans, Kaposi’s sarcoma, etc. (n=11). The study protocol was approved by the Ethics Committees of all participating hospitals, including Beijing Ditan Hospital, Chengdu Public Health, and Changsha First Hospital. Ultimately, the cohort comprised 375 HIV-positive patients with fractures, of whom 37 were excluded (**Figure 1**).

### Fasting blood sample collection and processing

Fasting blood samples were collected by venipuncture using Vacutainer tubes containing EDTA (Becton Dickinson, Franklin Lakes, NJ, USA) for flow cytometry analysis and morphological examination. Serum samples were allowed to stand for 45 minutes to clot prior to centrifugation. Next, the samples were centrifuged at 3000 g for 10 minutes. Subsequently, the serum aliquots were maintained in a cooled state, temperature controlled at -80°C.

### HIV diagnosis, HIV viral load measurement and T lymphocyte count

HIV diagnosis was established through the gold standard HIV-1/2 antibody testing, utilizing enzyme-linked immunoassay (ELISA) and rapid methods by laboratory doctors in our hospital. The 4th generation HIV kit (Abbott, UK), detection reagent: Murex HIV Ag/Ab, along with mini-VIDSA analyzer, Bio-Rad MODEL1575 plate washer, Axsym chemiluminescent immunoassay analyzer (Abbott, UK), and ELECYS2010 chameleon enzyme immunoassay apparatus (Roche, Switzerland) were employed.

Plasma viral load quantification was performed using the Abbott RealTime HIV viral load assay (m2000sp) from Abbott Molecular, IL, USA, with a sensitivity threshold of 40 copies/mL. Absolute CD4 cell counts in whole blood were obtained via standard flow cytometry using a Beckman Coulter Navios device (Beckman, San Jose, CA, USA).

Characterization of freshly isolated cell phenotypes and identification of T cell phenotypes utilized fluorochrome-tagged monoclonal antibodies supplied by BD Biosciences, San Jose, CA: anti-CD4 FITC (clone RPA-T4, RRID: AB_2562052) and anti-CD8AF700 (clone RPA-T8, RRID: AB_396953). These antibodies were incubated with the cells for 15 minutes at room temperature in the dark, followed by washing and analysis on a Beckman Coulter Navios flow cytometer (Beckman, San Jose, CA, USA). T helper and cytotoxic T cells were identified by their positive surface expression of CD4 and CD8, respectively, with their percentages reported relative to the gated total lymphocyte population.

### Definition of surgical site infections

Surgical site infections (SSIs) was the outcome variable in this study. The definition and classification of surgical site infection (SSI) was based on the American Academy of Orthopaedic Surgeon(AAOS) (Berríos-Torres et al., 2017; McLaren and Lundy, 2019): an infection related to surgery that occurs within 30 days after a surgery without an implant or within 1 year after a surgery with an implant (such as a fracture internal fixation system, vertebral pedicle screw, intervertebral fusion device, artificial intervertebral disc, prosthesis, etc.), including superficial incision infection, deep incision infection, and organ/tissue space infection. This clear definition allowed for the consistent identification of SSIs across all participating centers, facilitating the accurate assessment of SII’s predictive capability.

### Protocol for antibiotic prophylaxis

All patients included in this study received prophylactic antibiotics during surgery and for 48 hours postoperatively as per the institutional protocol. The antibiotics were administered intravenously, ensuring optimal tissue penetration and prophylaxis against surgical site infections. The specific antibiotics used were cefazolin or cefuroxime, a first-generation cephalosporin, which exhibits broad-spectrum activity effective against common surgical site pathogens. Administration of cefazolin or cefuroxime followed a strict dosing regimen: an intraoperative dose of 1.5 grams was given, followed by 1.5 grams every 8 hours (Q8h) postoperatively for 48 hours. This dosing and scheduling ensured therapeutic antibiotic levels during the perioperative period, vital for the prevention of surgical site infections.

### Potential risk factors associated with surgical site infections

To the best of our knowledge, we aimed to collect comprehensive data on potential risk factors for surgical site infections in HIV-positive patients. The gathered clinical information encompassed baseline demographics, HIV-related health indicators, comorbidity profiles, and procedure-related details. Specifically, demographic characteristics included age (<30 and ≥30 years), gender, HIV infection duration (<2 and ≥2 years), antiretroviral therapy (ART) status, and comorbidities such as HBV/HCV coinfection, and diabetes mellitus. Preoperative laboratory tests involved hemoglobin (Hb, <120 and ≥120 g/L), albumin (<40 and ≥40 g/L), CD4+ counts (<200 and ≥200 cells/ul), CD8+ counts (<400 and ≥400 cells/ul), HIV viral loads (<20,000 and ≥20,000 copies/ml), neutrophil counts, lymphocyte counts, and platelet counts. Surgical-related characteristics encompassed fracture site (upper limbs, lower limbs, and spine), fracture type (closed or open), anesthesia type (general or local), and blood loss (<400 and ≥400 ml). T lymphocyte subpopulations were detected using flow cytometry, which directly provided absolute CD4+ T lymphocyte counts or their relative proportions. Patient clinical characteristics were extracted from the hospital information system (HIS) medical record system, with data recorded and cross-checked by two or more residents.

### Selection of modeling features for surgical site infections

We initially considered a comprehensive set of potential predictors for surgical site infection (SSI) in HIV-positive fracture patients undergoing surgery. These candidate predictors were identified based on literature review and clinical expertise, and included patient demographics, HIV-related factors, comorbidities, surgical factors, and laboratory parameters.

To select the most predictive features for inclusion in the final model, we employed a systematic approach utilizing multiple analytical methods: 1. Univariate Logistic Regression Analysis: All potential predictors were first examined using univariate logistic regression. Variables with p-values < 0.05 were retained for further analysis. 2. Multivariate Logistic Regression Analysis: All potential predictors were then entered into a multivariate logistic regression model. Variables with p-values < 0.05 were retained for further analysis. 3. Support Vector Machine-Recursive Feature Elimination (SVM-RFE) Analysis: All potential predictors were also subjected to the SVM-RFE algorithm, an embedded method that iteratively removes the least important features during the model training process. 4. Comprehensive Evaluation: The results from the univariate analysis, multivariate analysis, and SVM-RFE were comprehensively evaluated, considering both statistical significance and clinical relevance.

The final set of predictive features included in the web-based risk calculator model for predicting SSI risk was determined through this systematic analysis process, which integrated findings from multiple analytical approaches and clinical considerations.

### The risk score formula for surgical site infection in HIV-positive patients

The risk of surgical site infection in HIV-positive fracture patients was modeled using multivariable logistic regression. The regression formula is given by: $\begin{array}{c} \text{logit}\left(\text{p}\right)={\text{β}}_{0}+{\text{β}}_{1}\times \text{HBV}\_\text{or}\_\text{and}\_\text{HCV}\left(\text{Yes or No}\right)+{\text{β}}_{2}\times \text{HIV}\_\text{RNA}\_\text{load}\\ (<20000\text{ copes or}\geq 20000\text{ copes})+{\text{β}}_{3}\times \text{Open\_fracture}\left(\text{Yes or No}\right)+{\text{β}}_{4}\times CD4\left(\text{cells}/\text{ul}\right)+{\text{β}}_{5}\\ \times \text{Neu}(10^{9}/\text{L})+{\text{β}}_{6}\times \text{Lym}\left(10^{9}/\text{L}\right) \end{array}$ The size and statistical significance of coefficients β1 to β6 reflect the size and importance of the effects of the respective variables.

Where $\text{logit}\left(\text{p}\right)=\ln\left(\frac{\text{p}}{1-\text{p}}\right)$ represents the log odds of surgical site infection, and the coefficients (β) were estimated as: $\begin{array}{c} \text{logit}\left(\text{p}\right)=-0.685+1.733\times \text{HBV\_or\_and\_HCV(Yes or No)}+1.028\times \text{HIV}\_\text{RNA}\_\text{load}\\ \left(<20000\text{ copes or}\geq 20000\text{ copes}\right)+1.337\times \text{Open\_fracture(Yes or No)}-0.006\times \text{CD4(cells/ul)}\\ +0.563\times \text{Neu}\left(10^{9}/\text{L}\right)-2.728\times \text{Lym(10\textasciicircum{}9/L)} \end{array}$ . 

This formula incorporates the following predictors: HBV/HCV co-infection, HIV RNA viral load, open fracture status, CD4+ T-cell count, neutrophil count, and lymphocyte count. The estimated regression coefficients quantify the impact of each predictor on the log odds of surgical site infection, after adjusting for the other variables in the model.

### Statistical analysis

We analyzed the data with the R version 4.1.3 software (https://www.R-project.org). If the P-value is less than 0.05, the difference is statistically significant. For continuous variables, we presented as mean ± standard deviation (SD) or the interquartile range (IQR) and use one-way ANOVA or Kruskal-Wallis U test for differences between two groups. Categorical variables were presented as percentages (n, %). The Chi-squared test and Fishers exact test were used to compare the differences between different groups. We used patients from Beijing Ditan Hospital as the training cohort and patients from Chengdu Public Health and Changsha First Hospital as the external validation cohort. Univariate, multivariate logistic regression analyses and SVM-RFE were performed to identify independent risk factors for SSIs. Variables with p-values < 0.05 were retained for further analysis. A web-based risk calculator was developed using the identified risk factors and validated using an external validation cohort. The dynamic model applications above were released based on this website https://www.shinyapps.io/. The performance of the nomogram was evaluated using the area under the receiver operating characteristic (AUC) curves, calibration plots, and decision curve analysis (DCA).

## Result

### Demographic characteristics and baseline of HIV-positive fracture patients with or without surgical site infection

In our study, 338 HIV-infected patients diagnosed with fractures were included. The overall baseline characteristics are summarized in **Table 1**. there were 226 (63.9%) patients from Beijing Ditan Hospital, 86 (10.6%) patients from Chengdu Public Health Clinical Medical Center and 36 (25.4%) patients from Changsha First Hospital. The overall SSI incidence was 10.7%. 36 (10.65%) were developing surgical site infections (SSIs) during the perioperative period.

**Table 1:** Baseline and demographic characteristics of HIV positive fracture patients in the training and testing groups.

Variable	Total	Train	Test	P-value
	*(N=338)*	*(N=216)*	*(N=122)*	
Source				<0.001
Beijing	216 (63.9%)	216 (100%)	0 (0.00%)	
Changsha	36 (10.7%)	0 (0.00%)	36 (29.5%)	
Chengdu	86 (25.4%)	0 (0.00%)	86 (70.5%)	
SSI				1.000
No	302 (89.3%)	193 (89.4%)	109 (89.3%)	
Yes	36 (10.7%)	23 (10.6%)	13 (10.7%)	
Gender (%)				0.199
Female	41 (12.1%)	22 (10.2%)	19 (15.6%)	
Male	297 (87.9%)	194 (89.8%)	103 (84.4%)	
Age (%, years)				0.011
<30	48 (14.2%)	39 (18.1%)	9 (7.38%)	
≥30	290 (85.8%)	177 (81.9%)	113 (92.6%)	
HBV or/and HCV (%)				0.808
No	267 (79.0%)	172 (79.6%)	95 (77.9%)	
Yes	71 (21.0%)	44 (20.4%)	27 (22.1%)	
Diabetes (%)				0.004
No	278 (82.2%)	188 (87.0%)	90 (73.8%)	
Yes	60 (17.8%)	28 (13.0%)	32 (26.2%)	
ART (%)				0.898
No	44 (13.0%)	29 (13.4%)	15 (12.3%)	
Yes	294 (87.0%)	187 (86.6%)	107 (87.7%)	
HIV infection duration				<0.001
<2_years	129 (38.2%)	126 (58.3%)	3 (2.46%)	
≥2_years	209 (61.8%)	90 (41.7%)	119 (97.5%)	
RNA load (%, copes/ml)				<0.001
<20000	293 (86.7%)	176 (81.5%)	117 (95.9%)	
≥20000	45 (13.3%)	40 (18.5%)	5 (4.10%)	
Bleed (%, ml)				<0.001
<400	231 (68.3%)	118 (54.6%)	113 (92.6%)	
≥400	107 (31.7%)	98 (45.4%)	9 (7.38%)	
Fracture Site (%)				0.097
Leg	238 (70.4%)	157 (72.7%)	81 (66.4%)	
Arm	66 (19.5%)	43 (19.9%)	23 (18.9%)	
Spine	34 (10.1%)	16 (7.41%)	18 (14.8%)	
Open_fracture (%)				0.797
No	316 (93.5%)	203 (94.0%)	113 (92.6%)	
Yes	22 (6.51%)	13 (6.02%)	9 (7.38%)	
Type_of_anesthesia				<0.001
RA	242 (71.6%)	137 (63.4%)	105 (86.1%)	
GA	96 (28.4%)	79 (36.6%)	17 (13.9%)	
CD4 (%, cells/ul)				0.465
<200	58 (17.2%)	40 (18.5%)	18 (14.8%)	
≥200	280 (82.8%)	176 (81.5%)	104 (85.2%)	
CD8 (%, cells/ul)				1.000
<400	50 (14.8%)	32 (14.8%)	18 (14.8%)	
≥400	288 (85.2%)	184 (85.2%)	104 (85.2%)	
CD4/CD8 Ratio (%)				0.073
<1.0	281 (83.1%)	186 (86.1%)	95 (77.9%)	
≥1.0	57 (16.9%)	30 (13.9%)	27 (22.1%)	
Albumin (g/L)	42.2 [39.1;46.2]	41.3 [38.5;45.9]	43.3 [40.4;46.7]	0.003
Hb (g/L)	137 [120;150]	135 [120;145]	144 [122;157]	0.001
Neu (10^9/L)	3.93 [2.84;5.50]	3.88 [2.83;5.36]	4.03 [2.87;5.84]	0.257
Lym (10^9/L)	1.56 [1.17;2.04]	1.51 [1.15;2.01]	1.62 [1.22;2.12]	0.219
Plt (10^9/L)	219 [179;267]	213 [176;272]	222 [184;263]	0.663

There were no significant differences in most baseline and demographic characteristics between patients who did and did not develop SSIs, including gender distribution (87.9% male, 12.1% female), age distribution (<30 years: 14.2%, ≥30 years: 85.8%), and duration of HIV infection (<2 years: 38.2%, ≥2 years: 61.8%). However, significant differences were observed in specific variables, notably hepatitis co-infection (HBV/HCV), HIV RNA load, and CD4+ T-cell count. Specifically, patients with hepatitis co-infection had a significantly higher incidence of SSI (P=0.010), as did patients with HIV RNA loads ≥20,000 Copes (P=0.001). Furthermore, patients with CD4+ T-cell counts <200 cells/ul had a significantly higher risk of developing SSIs compared to those with counts ≥200 cells/ul (P<0.001).

### Characteristics of the training and external validation cohorts

As shown in **Table 2**, comparing the training cohort (216 patients) with the external validation cohort (122 patients), we found similarities in most baseline characteristics across the two groups, but significant differences were observed in specific features. Notably, the training cohort comprised entirely patients from Beijing, while patients in the external validation cohort were from Chengdu and Changsha, aligning with our study’s design expectations (P<0.001). Additionally, significant differences between the cohorts were observed in age distribution (P=0.011), prevalence of diabetes (P=0.004), duration of HIV infection (P<0.001), HIV RNA load (P<0.001), intraoperative bleeding (P<0.001), and type of anesthesia used (P<0.001). These differences reflect the diversity in demographic characteristics and surgical-related factors among patients from different regions. Despite these differences, the overall incidence rates of SSIs did not show significant variation between the cohorts (P=1.000), validating the external applicability of our model.

**Table 2:** Baseline and demographic characteristics of HIV-positive fracture patients with and without surgical site infection.

Variables	[Total]	No SSI	Yes SSI	P-value
	*(N=338)*	*(N=302)*	*(N=36)*	
Source (%)				1.000
Beijing	216 (63.9%)	193 (63.9%)	23 (63.9%)	
Changsha	36 (10.7%)	32 (10.6%)	4 (11.1%)	
Chengdu	86 (25.4%)	77 (25.5%)	9 (25.0%)	
Gender (%)				0.415
Female	41 (12.1%)	35 (11.6%)	6 (16.7%)	
Male	297 (87.9%)	267 (88.4%)	30 (83.3%)	
Age (%, years)				1.000
<30	48 (14.2%)	43 (14.2%)	5 (13.9%)	
≥30	290 (85.8%)	259 (85.8%)	31 (86.1%)	
Hepatitis (%)				0.010
No	267 (79.0%)	245 (81.1%)	22 (61.1%)	
Yes	71 (21.0%)	57 (18.9%)	14 (38.9%)	
Diabetes (%)				0.330
No	278 (82.2%)	251 (83.1%)	27 (75.0%)	
Yes	60 (17.8%)	51 (16.9%)	9 (25.0%)	
ART (%)				0.797
No	44 (13.0%)	39 (12.9%)	5 (13.9%)	
Yes	294 (87.0%)	263 (87.1%)	31 (86.1%)	
HIV infection duration				0.316
<2_years	129 (38.2%)	112 (37.1%)	17 (47.2%)	
≥2_years	209 (61.8%)	190 (62.9%)	19 (52.8%)	
HIV RNA load (%)				0.001
<20000 (Copes)	293 (86.7%)	269 (89.1%)	24 (66.7%)	
≥20000 (Copes)	45 (13.3%)	33 (10.9%)	12 (33.3%)	
Bleed (%,ml)				0.239
<400	231 (68.3%)	210 (69.5%)	21 (58.3%)	
≥400	107 (31.7%)	92 (30.5%)	15 (41.7%)	
Fracture Site (%)				0.284
Leg	238 (70.4%)	213 (70.5%)	25 (69.4%)	
Arm	66 (19.5%)	61 (20.2%)	5 (13.9%)	
Spine	34 (10.1%)	28 (9.27%)	6 (16.7%)	
Open_fracture (%)				0.005
No	316 (93.5%)	287 (95.0%)	29 (80.6%)	
Yes	22 (6.51%)	15 (4.97%)	7 (19.4%)	
Type_of_anesthesia				0.374
RA	242 (71.6%)	219 (72.5%)	23 (63.9%)	
GA	96 (28.4%)	83 (27.5%)	13 (36.1%)	
CD4 (%, cells/ul)				<0.001
<200	58 (17.2%)	41 (13.6%)	17 (47.2%)	
≥200	280 (82.8%)	261 (86.4%)	19 (52.8%)	
CD8 (%,cells/ul)				1.000
<400	50 (14.8%)	45 (14.9%)	5 (13.9%)	
≥400	288 (85.2%)	257 (85.1%)	31 (86.1%)	
CD4/CD8 Ratio (%)				0.093
<1.0	281 (83.1%)	247 (81.8%)	34 (94.4%)	
≥1.0	57 (16.9%)	55 (18.2%)	2 (5.56%)	
Albumin (g/l)	42.2 [39.1;46.2]	42.3 [39.3;46.4]	40.0 [38.2;44.2]	0.014
Hb (g/L)	137 [120;150]	138 [122;151]	124 [111;141]	0.003
Neu (10^9/L)	3.93 [2.84;5.50]	3.83 [2.80;5.14]	5.77 [4.15;7.24]	<0.001
Lym (10^9/L)	1.56 [1.17;2.04]	1.62 [1.25;2.13]	0.98 [0.76;1.24]	<0.001
Plt (10^9/L)	219 [179;267]	219 [178;267]	217 [183;260]	0.917

### Univariate and multivariate logistic regression analysis

Our univariate logistic regression analysis identified several variables significantly associated with the risk of developing a surgical site infection (SSI) in HIV-positive fracture patients. Notably, HBV/HCV co-infection (OR = 2.90, 95% CI: 1.13–7.17, P=0.028), high HIV RNA load (OR = 5.18, 95% CI: 2.07–13.0, P<0.001), open fracture (OR = 6.42, 95% CI: 1.79–21.4, P=0.006), higher neu level (OR = 6.65, 95% CI: 2.55–17.2, P<0.001), lower albumin level (OR = 0.09, 95% CI: 0.04–0.24, P<0.001) and lower CD4+ T-cell counts (OR = 6.42, 95% CI: 1.79–21.4, P=0.006) were among the key factors with a significant association (**Table 3**).

**Table 3:** Univariate and multivariate binary logistic regression analysis of surgical site infection in HIV-positive fracture patients.

Variables	Univariate binary logistic regression		Multivariate binary logistic regression	
	OR (95% CI)	P-value	OR (95% CI)	P-value
Gender (%)		0.799		0.432
Female	—		—	
Male	1.21 (0.32, 7.94)		0.37 (0.03, 5.26)	
Age (%, years)		0.634		0.368
<30	—		—	
≥30	0.77 (0.28, 2.46)		0.43 (0.07, 2.87)	
HBV or/and HCV (%)		**0.028**		**0.014**
No	—		—	
Yes	2.90(1.13, 7.17)		7.41(1.59, 41.5)	
Diabetes (%)		0.520		0.213
No	—		—	
Yes	1.48(0.40, 4.36)		3.59(0.45, 27.9)	
ART (%)		0.459		0.104
No	—		—	
Yes	1.71(0.46, 11.1)		9.10(0.84, 190)	
HIV duration (%)		0.240		0.131
<2_year			—	
≥2_year	0.58(0.21, 1.42)		0.30(0.06, 1.32)	
HIV RNA load (%)		**<0.001**		0.120
<20000			—	
≥20000	5.18(2.07, 13.0)		3.33(0.73, 16.2)	
Bleed (%, ml)		0.115		0.712
<400	—		—	
≥400	2.02(0.84, 5.06)		1.32(0.30, 5.79)	
Fracture Site (%)		0.655		
Leg	—		—	
Arm	0.58(0.13, 1.82)		1.55(0.21, 9.87)	0.643
Spine	1.10(0.16, 4.39)		0.60(0.03, 7.43)	0.702
Open_fracture (%)		**0.006**		0.173
No	—		—	
Yes	6.42(1.79, 21.4)		4.70(0.51, 47.0)	
Type_of_anesthesia (%)		0.471		0.596
RA	—		—	
GA	1.38(0.56, 3.31)		1.48(0.34, 6.68)	
CD4 (%, cells/ul)		**<0.001**		**0.011**
<200	—		—	
≥200	0.13(0.05, 0.31)		0.11(0.02, 0.55)	
CD8 (%, cells/ul)		0.797		0.311
<400	—		—	
≥400	1.18(0.37, 5.23)		3.36(0.36, 43.6)	
CD4/CD8 Ratio		0.114		0.921
<1	—		—	
≥1	0.26(0.01, 1.30)		1.15(0.04, 14.2)	
Albumin (%, g/L)		**0.045**		0.734
<40	—		—	
≥40	0.41(0.17, 0.98)		0.77(0.16, 3.76)	
Hb (%, g/L)		0.113		0.536
<120	—		—	
≥120	0.47(0.19, 1.20)		1.74(0.32, 11.3)	
Neu (%, 10^9/L)		**<0.001**		**0.007**
<6.3	—		—	
≥6.3	6.65(2.55, 17.2)		9.65(1.99, 57.4)	
Lym (%,10^9/L)		**<0.001**		**<0.001**
<1.1	—		—	
≥1.1	0.09(0.04, 0.24)		0.05(0.01, 0.25)	
Plt (%, 10^9/L)		0.121		0.102
<200	—		—	
≥200	2.09(0.83, 6.01)		4.32(0.85, 30.1)	

OR, Odds Ratio; CI, Confidence Interval.Black bold font means that the P-value is statistically significant.

The multivariate logistic regression analysis, which adjusted for all potential confounders, further refined these findings (**Table 3**). It revealed that hepatitis co-infection (OR = 7.41, 95% CI: 1.59–41.5, P=0.014), and low CD4+ T-cell count (<200 cells/μl, OR = 0.11, 95% CI: 0.02–0.55, P=0.011) remained significantly associated with an increased risk of SSI. High neutrophil count (Neu) and low lymphocyte count (Lym) were also identified as significant predictors of SSI risk in the final model (Neu: OR = 9.65, 95% CI: 1.99–57.4, P=0.007; Lym: OR = 0.05, 95% CI: 0.01–0.25, P<0.001).

### Model feature selection using SVM-RFE algorithm and final model performance for risk of SSIs in in training cohort

After svm-RFE analysis (**Supplementary Table 1**; **Figure 1**), it was found that we obtained the highest accuracy by modeling with three variables (Neu, CD4, lym). We evaluated univariate, multivariate, and SVM-RFE results together, i.e., after considering statistical significance and clinical relevance, we identified six independent risk factors and included them in a web-based risk calculator: HBV/HCV coinfection, HIV RNA load, CD4 + T cell count, neutrophil count, and lymphocyte count.

The risk of surgical site infection in HIV-positive fracture patients was modeled using multivariable logistic regression. To assess the overall model fit, several metrics were calculated: P-values: 1) The p-values for the predictor variables HBV_or_and_HCV, CD4, Neu, and Lym were less than 0.05, indicating their statistical significance in predicting surgical site infection. 2) Pseudo-R² (McFadden’s R²): McFadden’s pseudo-R² value for the model was 0.493, suggesting that the model explains approximately 49.3% of the variation in the outcome variable using the included predictors. 3) Likelihood Ratio Test: The likelihood ratio test comparing the full model with the null model (intercept-only) yielded a χ² statistic of 72.128 (p-value < 0.001), indicating that the inclusion of the predictor variables significantly improves the model’s fit. These metrics demonstrate that the logistic regression model has good explanatory power and representativeness in predicting surgical site infection risk in HIV-positive fracture patients undergoing surgery.

### Clinical application and performance of the risk calculator in training and external validation cohort

**Figure 2:**
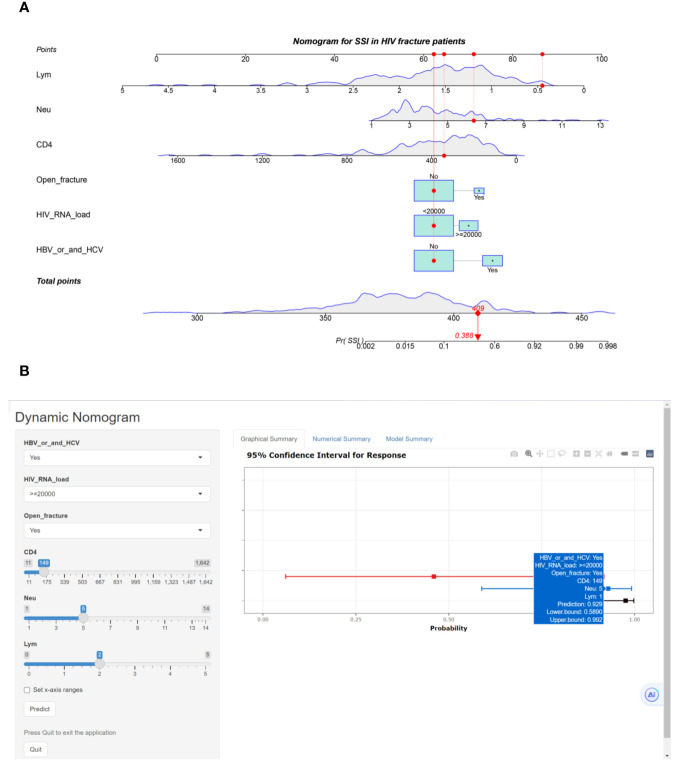
**(A)** Classical nomogram predicting surgical site infection in HIV-positive fracture patients; **(B)** Web-based risk calculator interface predicting surgical site infection in HIV-positive fracture patients.

We used six risk factors to construct a diagnostic nomogram to assess the risk score for surgical site infection in HIV-positive fracture patients (**Figure 2A**). Furthermore, the web-based risk calculator is publicly available at https://sydtliubo.shinyapps.io/DynNom_for_SSI/, allowing users to easily calculate the risk of SSI for individual HIV-positive fracture patients based on their specific risk factor profiles (**Figure 2B**).

**Figure 3:**
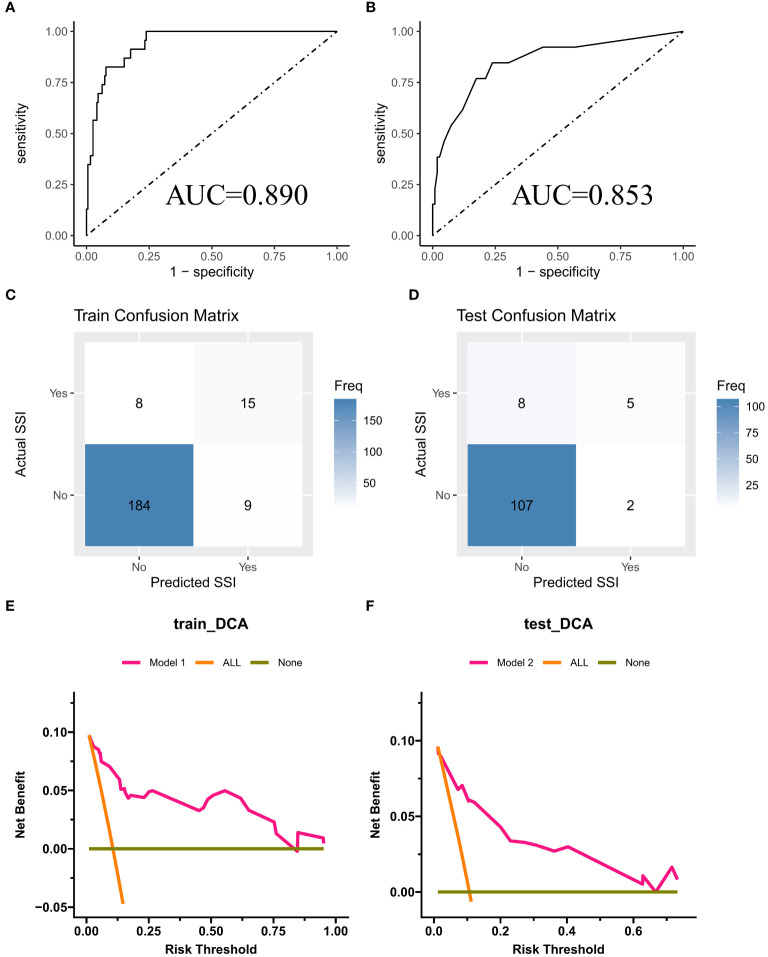
**(A, B)** ROC curves for training cohort and external validation cohort; **(C, D)** Confusion Matrix Plot for Training Set and External Validation Set; **(E, F)** DCA curves for training cohort and external validation cohort.

**Figure 4:**
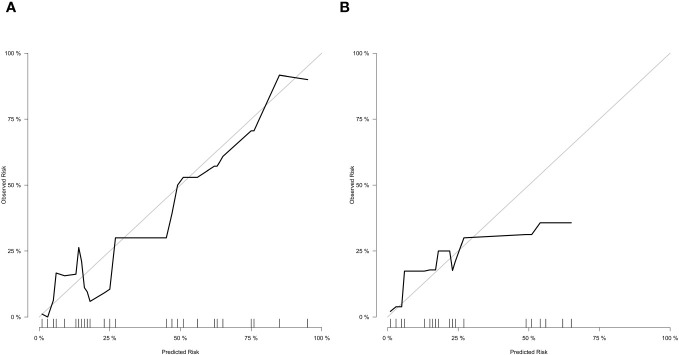
Calibration curves for training cohort **(A)** and external validation cohort **(B)**.

In the training cohort (**Figure 3A**), the nomogram demonstrated good discrimination, with an AUC of 0.890 (95% CI: 0.845–0.935). The confusion matrix for the training cohort is presented in **Figure 3C**, showing a sensitivity of 0.9534, specificity of 0.6522, positive predictive value of 0.9583, and negative predictive value of 0.6250. In the external validation cohort (**Figure 3B**), the AUC was 0.853 (95% CI: 0.799–0.907), indicating good predictive performance. The confusion matrix for the validation cohort is presented in **Figure 3D**, revealing a sensitivity of 0.9817, specificity of 0.3846, positive predictive value of 0.9304, and negative predictive value of 0.7143. The calibration plots showed good agreement between predicted and observed SSI probabilities in both cohorts (**Figures 4A, B**). Decision curve analysis (DCA) revealed that the nomogram had clinical utility across a wide range of threshold probabilities, outperforming the treat-all and treat-none strategies (**Figures 3E, F**).

## Discussion

In our multicenter retrospective cohort study, we included a total of 338 HIV-positive patients who underwent fracture surgery, with 216 patients in the training cohort and 122 patients in the external validation cohort. The overall incidence of surgical site infections (SSIs) was found to be 10.7%. This finding highlights the substantial burden of SSIs among HIV-positive fracture patients in China, underscoring the need for effective risk prediction and preventive strategies. Through univariate and multivariate logistic regression analyses, as well as the application of the SVM-RFE method, we identified six independent risk factors for SSIs in this patient population. These factors included HBV and/or HCV co-infection, HIV RNA load, CD4+ T-cell count, and neutrophil (Neu) and lymphocyte (Lym) levels. The identification of these key risk factors not only provides valuable insights into the pathogenesis of SSIs in HIV-positive fracture patients but also forms the basis for the development of our web-based risk calculator. The incorporation of these risk factors into the nomogram allows for personalized risk prediction, enabling clinicians to identify high-risk patients and implement targeted preventive measures. Moreover, the web-based platform ensures the nomogram’s accessibility and ease of use, facilitating its integration into clinical decision-making processes. In summary, our study reveals a considerable incidence of SSIs among HIV-positive fracture patients in China and identifies six key risk factors that contribute to SSI development. These findings lay the foundation for the subsequent development and validation of a novel web-based risk calculator, which holds promise for improving SSI risk prediction and guiding clinical management in this vulnerable patient population.

Previous studies have confirmed that HIV infection was associated with an increased risk of surgical complications, including SSIs. For instance, a systematic review and meta-analysis by Kigera et al. (2012) found that HIV-positive patients had a significantly higher risk of postoperative SSIs compared to HIV-negative patients (Kigera et al., 2012). Similarly, a retrospective cohort study by Horberg et al. (2006) demonstrated that HIV-infected patients had a higher incidence of SSIs following surgical procedures (Horberg et al., 2006). These findings underscore the importance of considering HIV status when assessing surgical risk and developing preventive strategies. However, the incidence of SSIs in HIV-positive patients undergoing fracture surgery remains understudied. Regarding risk factors, a meta-analysis by Fan et al. (2014) identified several patient-related and procedure-related factors associated with SSI development, such as diabetes, smoking, and prolonged operative time (Fan et al., 2014). A systematic review by Xing et al. (2013) revealed that the pooled incidence of SSI in HIV-positive patients was 12.1% (95% CI: 8.6%-16.7%), highlighting the substantial burden of this complication (Xing et al., 2013). Several risk factors have been consistently identified across studies, including low CD4 count (<200 cells/ul), high viral load (>1000 copies/mL), advanced HIV disease stage, and malnutrition (Chen et al., 2009; Namba et al., 2013). Other reported risk factors include prolonged operative time, contaminated wound classification, and emergent surgical procedures (Kigera et al., 2012; Zhang et al., 2012). Nevertheless, the specific risk factors for SSIs in HIV-positive fracture patients warrant further investigation. Moreover, there is a paucity of SSI risk prediction models specifically designed for HIV-positive fracture patients. The development of a user-friendly, web-based risk calculator tailored to this patient population would address these limitations and provide a valuable tool for personalized risk assessment.

Our study identified HBV and/or HCV co-infection as a significant risk factor for SSIs in HIV-positive fracture patients. This finding is consistent with previous research suggesting that co-infection with viral hepatitis may compromise immune function and increase susceptibility to infections (Matthews and Rockstroh, 2011). The mechanism underlying this association may involve the complex interplay between viral infections and the immune system. Chronic viral hepatitis can lead to liver dysfunction, which may impair the production of immune mediators and compromise the body’s ability to mount an effective immune response against bacterial pathogens (Hernandez and Sherman, 2011). Moreover, co-infection with HIV and viral hepatitis may result in accelerated disease progression and further immune dysregulation (Boulougoura and Sereti, 2016). We found that higher HIV RNA load was associated with an increased risk of SSIs in HIV-positive fracture patients. This observation is in line with previous studies demonstrating that uncontrolled HIV replication and high viral load are associated with immune dysfunction and increased susceptibility to infections (Deeks et al., 2004). High HIV RNA load may impair the function of immune cells, such as CD4+ T cells and natural killer cells, which play crucial roles in the defense against bacterial infections (Leite et al., 2023; Dauby et al., 2024). Furthermore, high viral load may contribute to a pro-inflammatory state, which can disrupt the normal healing process and increase the risk of SSIs. The lower CD4+ T-cell count was a significant risk factor for SSIs in HIV-positive fracture patients. This finding is consistent with the well-established role of CD4+ T cells in the immune response against bacterial infections (Wan, 2010). CD4+ T cells orchestrate the adaptive immune response by activating and coordinating the functions of other immune cells, such as B cells and macrophages. In HIV infection, the progressive depletion of CD4+ T cells leads to immunodeficiency and increased susceptibility to opportunistic infections (Okoye and Picker, 2013). Low CD4+ T-cell count has been associated with an increased risk of SSIs in various surgical populations, highlighting the importance of this factor in predicting SSI risk (Wijesekera et al., 2016).

Our study identified neutrophil (Neu) and lymphocyte (Lym) levels as independent risk factors for SSIs in HIV-positive fracture patients. Neutrophils are key components of the innate immune system and play a crucial role in the early defense against bacterial infections (Kolaczkowska and Kubes, 2013). Lymphocytes, including CD4+ T cells and B cells, are essential for the adaptive immune response and the development of immunological memory (Bonilla and Oettgen, 2010). Alterations in Neu and Lym levels may reflect underlying immune dysfunction and increased susceptibility to infections. For instance, neutropenia, a condition characterized by low neutrophil count, has been associated with an increased risk of SSIs in various surgical settings (Chu et al., 2005), our findings suggest that a higher neutrophil count may also indicate an elevated risk. This may be due to the fact that a higher neutrophil count often signifies a heightened inflammatory state in the patient, which can predispose them to an increased risk of perioperative surgical site infections. Similarly, lymphopenia, particularly CD4+ T-cell depletion, has been linked to an increased risk of postoperative infections in HIV-positive patients (Paiardini and Müller-Trutwin, 2013). It is important to acknowledge that the risk of SSIs in HIV-positive fracture patients is likely influenced by the complex interplay of multiple factors. The identified risk factors, such as HBV/HCV co-infection, HIV RNA load, CD4+ T-cell count, and Neu and Lym levels, may have synergistic effects on SSI risk. For instance, the combination of high HIV RNA load and low CD4+ T-cell count may result in a more profound immune dysfunction and a higher risk of SSIs compared to either factor alone (Coombes and Gregory, 2019). Similarly, the presence of HBV/HCV co-infection in the setting of uncontrolled HIV replication may further exacerbate immune impairment and increase SSI risk (Ratnakar et al., 2024). Understanding the interplay of these risk factors is crucial for developing comprehensive risk assessment tools and targeted preventive strategies.

This study introduces the first web-based risk calculator specifically designed for predicting SSI risk in HIV-positive fracture patients. This innovative tool addresses the lack of user-friendly, accessible risk prediction models tailored to this vulnerable patient population. By leveraging advanced web technologies, we have developed a platform that allows clinicians to input patient-specific data and obtain personalized SSI risk estimates instantly. This web-based approach eliminates the need for manual calculation and facilitates the integration of the nomogram into clinical workflows, thereby enhancing its practical utility. A key strength of our study lies in its large-scale, multicenter design. We included a total of 338 HIV-positive fracture patients from three tertiary hospitals in China, with 216 patients in the training cohort and 122 patients in the validation cohort. This multicenter approach ensures the representativeness of our study population and enhances the generalizability of our findings. The large sample size also increases the statistical power of our analyses and allows for more robust conclusions. In addition to the conventional univariate and multivariate logistic regression analyses, we employed a novel risk factor selection method, namely support vector machine-recursive feature elimination (SVM-RFE). This machine learning technique combines the advantages of support vector machines (SVMs) and recursive feature elimination (RFE) to identify the most informative risk factors for SSI development. The SVM-RFE iteratively eliminates less relevant features while retaining the most discriminative ones, resulting in a more parsimonious and accurate risk prediction model. The application of this advanced technique in our study represents a methodological innovation in the field of SSI risk prediction and showcases the potential of machine learning in enhancing clinical decision support tools.

There are also limitations to our study. One limitation of our study is its retrospective design. Retrospective studies are prone to selection bias, as the inclusion of patients is based on the availability of complete medical records and follow-up data. However, we attempted to mitigate these biases by employing strict inclusion and exclusion criteria and by carefully reviewing and validating the collected data. Additionally, all patients received prophylactic antibiotics, the study did not collect detailed data on the specific antibiotics used, their dosing, or the exact timing of administration. These factors could potentially influence the incidence of SSI and should be more rigorously controlled and documented in future studies. Another limitation is the potential presence of unmeasured confounders, such as socioeconomic status, nutritional status, or adherence to antiretroviral therapy, which may have an impact on the observed associations. Furthermore, while our study included an external validation cohort from two additional hospitals, the generalizability of our findings to other populations and healthcare settings may be limited. The performance of the web-based risk calculator should be further validated in larger, more diverse patient populations to assess its applicability in different contexts. Additionally, our current risk calculator incorporates only six risk factors, and future research should explore additional relevant risk factors and biomarkers to potentially improve the model’s predictive performance.

To address these limitations, future prospective, multicenter studies are needed to further validate and refine the predictive model. For high-risk patients, we need to re-evaluate the guidelines for antibiotic prophylaxis and tailor them according to specific risk factors. Moreover, for non-urgent surgical cases, we could consider postponing surgery based on the patient’s risk factors to reduce the incidence of surgical site infections. By implementing these measures, future studies can further optimize and refine this risk assessment tool, providing valuable guidance for risk stratification and clinical decision-making in HIV-positive fracture patients undergoing surgery. Despite these limitations, our study has several notable strengths. The multicenter design, large sample size, and rigorous statistical analyses contribute to the robustness of our findings. The development of a user-friendly, web-based platform for SSI risk prediction addresses the need for accessible and personalized clinical decision support tools. Moreover, the excellent performance of the nomogram highlights its potential to improve risk stratification and guide preventive strategies in HIV-positive fracture patients. While the current model effectively captures linear relationships between risk factors and SSIs, future research could explore the application of machine learning methods, such as random forests, to train the model. These advanced techniques have the potential to capture non-linear relationships, which may further improve prediction accuracy. However, this approach also poses challenges, including the need for larger datasets, more complex model training, and potential overfitting issues. Balancing these considerations will be crucial in determining the most effective modeling approach for predicting SSIs.

To ensure the effective integration of our risk calculator into clinical workflows, especially in resource-limited settings where its application could be most impactful, we are focusing on several key areas. Firstly, we are committed to developing a user-friendly interface accessible through web or mobile platforms, enabling clinicians to efficiently input patient data and receive rapid risk assessments. We also recognize the importance of adapting the calculator to local contexts, and thus, we plan to undertake targeted validation and calibration exercises to maintain its relevance and accuracy within specific populations. Additionally, seamless integration with pre-existing health information systems is crucial, which is why we’ll collaborate closely with local partners for smooth implementation. To empower clinicians to fully utilize the calculator, comprehensive training sessions will be conducted, focusing on its effective usage and interpretation of results. Lastly, sustainability is key, and we’ve established mechanisms for ongoing support, updates, and maintenance, ensuring the calculator remains a valuable clinical tool over time. By adopting this comprehensive approach, we aim to seamlessly integrate our risk calculator into clinical workflows, thereby enhancing patient outcomes, especially in resource-constrained environments.

## Conclusion

In conclusion, our study introduces a novel web-based risk calculator for predicting SSI risk in HIV-positive fracture patients. This user-friendly tool incorporates key risk factors, including HBV/HCV co-infection, HIV RNA load, CD4+ T-cell count, and Neu and Lym levels, to provide personalized risk estimates. The nomogram demonstrated excellent performance in the training and external validation cohorts, highlighting its potential to guide clinical decision-making and improve patient outcomes. The development of this web-based platform addresses the need for accessible and personalized risk prediction tools in this vulnerable patient population. By enabling individualized risk assessment and targeted preventive strategies, the nomogram has the potential to optimize perioperative management, inform patient counseling, and facilitate quality improvement initiatives. Future research should focus on prospective multicenter validation, incorporation of additional risk factors to further enhance the nomogram’s accuracy and clinical utility. With continued refinement and validation, this web-based risk calculator has the potential to revolutionize SSI risk prediction and prevention in HIV-positive fracture patients, ultimately improving patient care and outcomes.

## Data availability statement

The original contributions presented in the study are included in the article/**Supplementary Material**. Further inquiries can be directed to the corresponding author.

## Ethics statement

All patients provided informed consent for publication of our study and accompanying images. The Ethics Committee of the Beijing Ditan Hospital of Capital Medical University approved the study.

## Author contributions

BL: Writing – original draft, Writing – review & editing. WG: Writing – review & editing. LH: Writing – review & editing. QZ: Writing – review & editing.

## Supplementary material

Supplementary Figure 1 Accuracy line plot when different latent variables are included in the model in SVM-RFE analysis.
