# Supplementary material for: A novel web-based risk calculator for predicting surgical site infection in HIV-positive facture patients: a multicenter cohort study in China
Source: Front Cell Infect Microbiol. 2024 Jun 26;14:1408388. doi: 10.3389/fcimb.2024.1408388 (PMC11233529; doi:10.3389/fcimb.2024.1408388)
Supplement: Supplementary file 1 [file DataSheet_1.docx]

**Supplementary**

**Table 1** The results of Resampling performance over subset size using Recursive feature selection after Outer resampling method: Bootstrapped (25 reps).

| Variables | **Accuracy** | **Kappa** | **AccuracySD** | **KappaSD** | **Selected** |
| --- | --- | --- | --- | --- | --- |
| 1 | 0.8872 | -0.004747 | 0.03592 | 0.03207 |  |
| 2 | 0.8929 | 0.070602 | 0.02721 | 0.16344 |  |
| 3 | 0.8982 | 0.07373 | 0.02499 | 0.14175 | * |
| 4 | 0.8864 | 0.161263 | 0.02294 | 0.14117 |  |
| 5 | 0.8886 | 0.134505 | 0.02382 | 0.14379 |  |
| 6 | 0.8903 | 0.138574 | 0.02068 | 0.15065 |  |
| 7 | 0.8932 | 0.113812 | 0.01943 | 0.14866 |  |
| 8 | 0.8907 | 0.086786 | 0.01709 | 0.13784 |  |
| 9 | 0.8861 | 0.129237 | 0.01764 | 0.16337 |  |
| 10 | 0.8887 | 0.116758 | 0.01784 | 0.14421 |  |
| 11 | 0.8903 | 0.130062 | 0.02179 | 0.13672 |  |
| 12 | 0.8935 | 0.151185 | 0.02361 | 0.16584 |  |
| 13 | 0.8939 | 0.122434 | 0.02313 | 0.14983 |  |
| 14 | 0.8938 | 0.105284 | 0.02393 | 0.15178 |  |
| 15 | 0.895 | 0.110019 | 0.02543 | 0.13488 |  |
| 16 | 0.8924 | 0.134634 | 0.02509 | 0.13667 |  |
| 17 | 0.8913 | 0.120899 | 0.02591 | 0.14638 |  |
| 18 | 0.893 | 0.108372 | 0.02245 | 0.13002 |  |
| 19 | 0.8959 | 0.131421 | 0.02717 | 0.16236 |  |

The top 3 variables (out of 3): Lym, CD4, Neu
