# Supplementary figures and images for: A novel web-based risk calculator for predicting surgical site infection in HIV-positive facture patients: a multicenter cohort study in China
Source: Front Cell Infect Microbiol. 2024 Jun 26;14:1408388. doi: 10.3389/fcimb.2024.1408388 (PMC11233529; doi:10.3389/fcimb.2024.1408388)

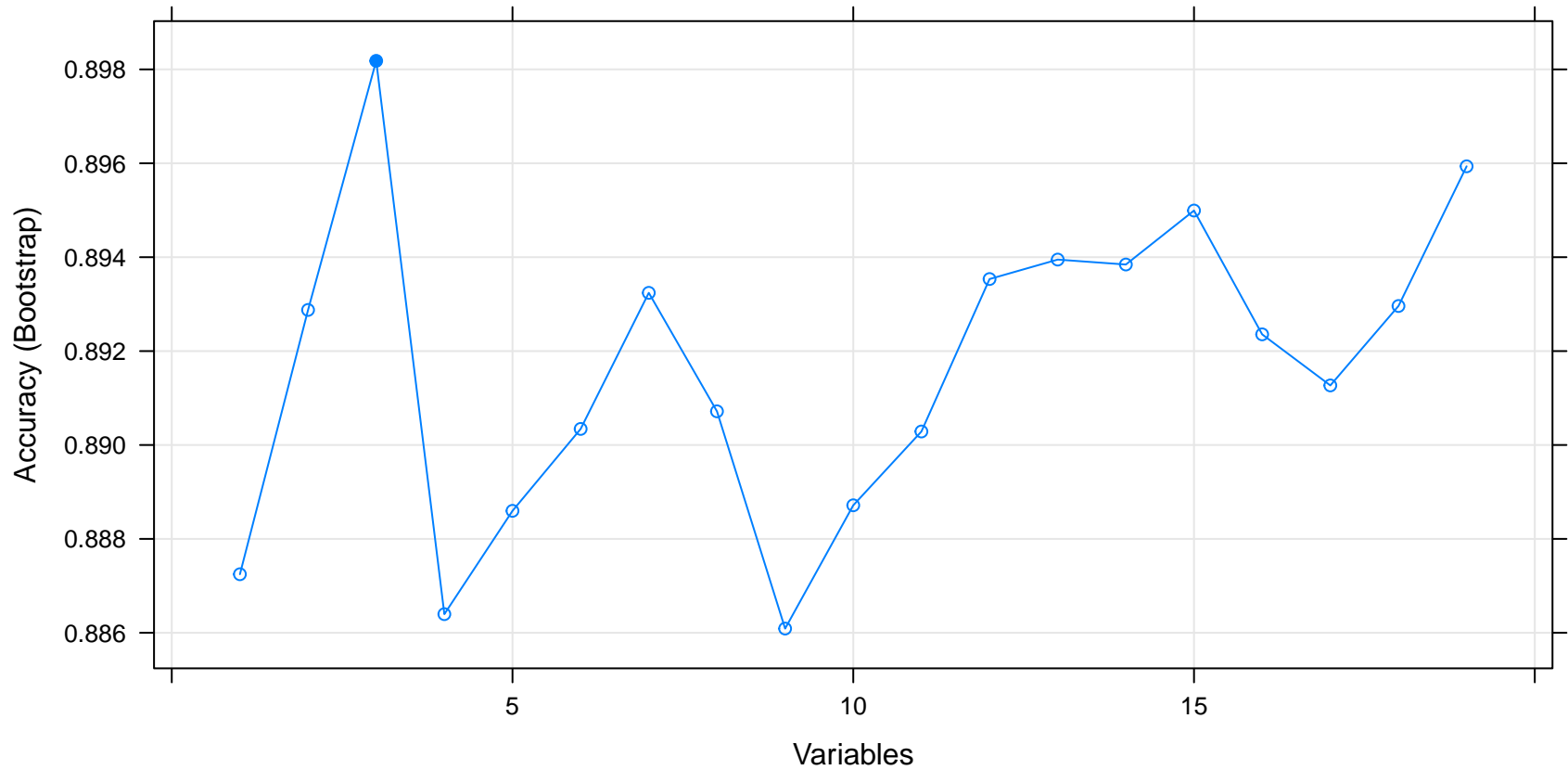

Supplement: Supplementary Figure 1 — Accuracy line plot when different latent variables are included in the model in SVM-RFE analysis. [file Image_1.pdf]
